# Sulforaphane Treatment in Children with Autism: A Prospective Randomized Double-Blind Study

**DOI:** 10.3390/nu15030718

**Published:** 2023-01-31

**Authors:** Martin Magner, Kateřina Thorová, Veronika Župová, Milan Houška, Ivana Švandová, Pavla Novotná, Jan Tříska, Naděžda Vrchotová, Ivo Soural, Ladislav Jílek

**Affiliations:** 1 Department of Paediatrics and Inherited Metabolic Disorders, First Faculty of Medicine, Charles University, General University Hospital, 128 08 Prague 2, Czech Republic; 2 National Institute for Autism, 180 00 Prague 8, Czech Republic; 3 Food Research Institute Prague, 102 00 Prague 10, Czech Republic; 4 Global Change Research Institute CAS, 603 00 Brno, Czech Republic; 5 Faculty of Horticulture, Mendel University in Brno, 691 44 Lednice, Czech Republic; 6 Pure Food Norway, 1400 Ski, Norway

**Keywords:** autism, sulforaphane, children

## Abstract

Autism spectrum disorder (ASD) is a pervasive neurodevelopmental disorder with repetitive behaviour which affects interaction and communication. Sulforaphane (SFN), an isothiocyanate abundant in the seeds and sprouts of cruciferous vegetables, has been shown to be effective in alleviating autistic behaviour. We performed a prospective double-blind placebo-controlled study to examine the possible effect of SFN in a paediatric cohort aged three to seven years based on measurements of the Autism Diagnostic Observation Schedule-2 (ADOS-2), the Social Responsiveness Scale-2 (SRS-2), and the Aberrant Behaviour Checklist (ABC). The study consisted of three visits over the duration of 36 weeks (baseline, 18 weeks, and 36 weeks). Twenty-eight of the 40 randomized children completed the study. The mean total raw scores on ABC and SRS-2 improved in both groups, but none of the changes reached statistical significance (ABC: 0 weeks *p* = 0.2742, 18 weeks *p* = 0.4352, and 36 weeks 0.576; SRS-2: 0 weeks *p* = 0.5235, 18 weeks *p* = 0.9176, and 36 weeks 0.7435). Changes in the assessment of the ADOS-2 subscale scores also did not differ between the two study cohorts (ADOS-2: 0 weeks *p* = 0.8782, 18 weeks *p* = 0.4788, and 36 weeks 0.9414). We found no significant clinical improvement in the behavioural outcome measures evaluated in children with ASD aged 3–7 years that were treated with sulforaphane.

## 1. Introduction

Autism spectrum disorder (ASD) is a pervasive neurodevelopmental disorder affecting the ability to initiate and sustain reciprocal social interaction and communication, as well as being characterized by repetitive behaviour, interests, or activities [1]. Many factors are associated with an increased likelihood of developing ASD; however, no definite causes have been established yet. A complex setting of interactions in the developing brain is closely linked to synaptic signalling and gene expression. A subtle genetic change can lead to synaptic changes relating to a hallmark ASD symptom. The estimated ASD heritability ranges approximately from 40% to 90%. More than 100 genes and genomic regions have now been confidently associated with ASD. Most of them are represented by heterozygous, germline, and de novo mutations. The extent of genetic changes varies in size from single-nucleotide variants to copy-number variations (CNVs) [2]. Most theories of ASD pathophysiology refer to neurodevelopmental disorders in general and ASD specificity still remains needed; no crystallized clinical biomarkers for ASD have been established [3,4]. Moreover, no cure for ASD is currently available; however, individuals with ASD can benefit from an individualized combination of treatment and services [5].

Even though the exhaustive pathogenesis description of ASD remains masked, a substantial body of publications has indicated that the pathophysiology of ASD is affected by immune response dysregulation, inflammatory conditions, oxidative stress, and mitochondrial dysfunction [6,7]. Children with ASD show abnormal oxidative stress patterns in the peripheral and brain tissues [7,8]. The activity and metabolic pathways of several inflammatory-response enzymes in the brain tissue of ASD patients were shown to be reduced. Several studies demonstrated inflammation dysregulation in ASD patients, either within the central nervous system or the periphery. Abnormal alterations in microglial cell activation, altered proinflammatory cytokine production, and immune-related gene expression have been documented in children with ASD [9,10]. A growing number of papers also indicate that an interaction between oxidative stress and mitochondrial function may play a role in the pathogenesis of ASD [11]. Biomarkers of mitochondrial dysfunction were reported to be associated with autistic behaviour and ASD severity [10].

Sulforaphane (SFN, 4-methylsulfinylbutyl isothiocyanate) is an isothiocyanate abundant in the seeds and sprouts of cruciferous vegetables. This multifunctional phytochemical is beneficial for cytoprotection, antioxidant and anti-inflammatory responses, mitochondrial and synaptic function, neuroinflammation, and neuroprotective mechanisms [9,12]. Additionally, numerous in vitro studies, animal models, and various clinical studies have depicted the beneficial effects of SFN [13].

The hope for the SFN’s efficiency in autism was brought to light by the placebo-controlled double-blind randomized study of Singh et al. [14] which included young men (aged 13–27; 29 SFN/15 placebo) who showed significant improvement in the Aberrant Behaviour Checklist (ABC), the SRS-2, and Clinical Global Impression (CGI-I) scores with improved social interaction, verbal communication, and a reduction in abnormal behaviour. Several other case reports and open-label studies have indicated the favourable effects of SFN [15,16]. Evans and Fuller [15] studied the extent of improvement in ASD patients taking SFN. In a limited number of patients followed for 28 weeks, the authors found that 74 (80%) of 92 attributes improved, with significant improvements in 36 (39%) of the attributes. Moreover, the improvements lasted for an interval of 28 weeks [15]. Similarly, in an open-label study by Bent et al. [16], clinical testing using ABC and SRS scores in 15 children confirmed significant improvement in ABC and SRS scores. However, these promising results were not confirmed in other placebo-controlled studies, e.g., in children with autism (22 SFN/23 placebo) leading to improvement in ABC, but not in the total and all subscale scores of the primary outcome measures—the Ohio Autism Clinical Impressions Scale (OACIS) and SRS-2 [17]. Additionally, only nonsignificant improvement of the primary outcome (SRS-2) was observed in young men aged 13–30 years old with moderate to severe autism spectrum disorder that were treated with SFN (24 SFN/24 placebo) (NCT0290995). Data from more extensive trials have not yet been published [18,19].

As young children with diagnosed autism are believed to be sensitive to any possible therapeutic intervention, we aimed to examine the possible effect of SFN in the age cohort of three to seven years in the present study by using the objective and subjective criteria for mental and behavioural changes (ADOS-2, SRS-2 and ABC).

## 2. Patients and Methods

### 2.1. Patients and Study Design

Children aged 3–7 years with a confirmed clinical diagnosis of ASD that was reconfirmed based upon the DSM-5 symptom checklist were enrolled in this double-blind prospective study. Inclusion criteria were an ASD diagnosis, no prior use of sulforaphane-containing supplements, completion of all follow-ups, and a parent/caregiver willing to consent. Exclusion criteria involved proven genetic diagnosis (secondary autism), seizure disorder, chronic disease, and medication affecting testing.

The study comprised three visits. The first visit included screening, randomization, and the start of treatment. Two follow-ups were performed at 18 and 36 weeks after the first visit. At each visit, the medical history, physical examination including vital signs, blood samples, adverse event reporting, and SRS-2, ABC, and ADOS-2 scores were obtained. Treatment was discontinued after the 36-week visit. The study was carried out in a randomized, double-blind, placebo-controlled manner. Parents/caregivers were then informed whether their child received sulforaphane or placebo.

SFN was administered orally once a day. The subjects were dosed uniformly with 50 µmol SFN per day. Parents/caregivers were instructed to mix the content of one PE/AL bag (1.2 g of the treatment) with a small amount of cold water or other cold food (yogurt, fruit juice). The placebo group received an equivalent dose of spinach puree powder.

### 2.2. Psychological Examination

To evaluate the severity of ASD symptoms and their changes within the study period, the standardized ADOS-2 assessment was delivered. The ADOS-2 assessments were videotaped and cross-coded by two trained psychologists. All researchers were masked to treatment group assignment. Monitored items were selected from developmentally staged ADOS-2 modules (Module 1—early development nonverbal; Module 1—early words; Module 2—phrase speech; and Module 3—fluent speech). We chose this autism diagnostic symptom measurement instrument because it was possible to rate it in a masked manner. We were able to assess different core symptoms of autism because of its excellent interpersonal objectivity (interrater reliability).

Two parent- or caregiver-reported questionnaires (ABC and SRS-2) were collected at each visit.

#### 2.2.1. ADOS-2

ADOS-2 is a standard, semistructured diagnosis assessment of a range of activities focusing on reciprocal social interactions, communication and language, play, and restricted and repetitive, stereotyped interests and behaviours [20].

#### 2.2.2. SRS-2

SRS-2 is a norm-referenced quantitative assessment measuring 65 items about children’s behaviour. Parents/caregivers rate five subdomains (social awareness, social cognition, social communication, social motivation subdomains, and restricted, repetitive behaviors) [21]. Total raw scores were compared.

#### 2.2.3. ABC

ABC, a secondary rating scale, was originally developed to assess the effectiveness of psychotropic medication and measures the severity of a range of behaviour problems across five subscales. At each follow-up, parents/caregivers evaluated subdomains as follows: irritability, lethargy/social withdrawal, stereotypic behaviour, hyperactivity/noncompliance, and inappropriate speech. Summary scores were used for psychometric analysis.

### 2.3. Preparation of Sulforaphane-Rich Broccoli Sprouts and Placebo Powder

A sulforaphane-rich broccoli sprout/red radish sprout powder mix was prepared using BroccoPhane^®^, a broccoli sprout powder standardized for sulforaphane (Bioriginal, Den Bommel, The Netherlands, Europe), and radish sprout powder produced in local laboratories. In short, young red radish sprouts harvested at their nutritional peak were kept at −18 °C overnight, divided into aluminium dishes, lyophilized, and powdered. Broccoli and red radish sprout powder were mixed in a 9:1 ratio. The mix was kept under constant stirring at 100 °C for 40 min, cooled, and mixed with pure water (45 °C) in a 1:10 ratio. The suspension was brought to 100 °C, stirred for 10 min, subsequently cooled to 20 °C, and divided into aluminium dishes in 0.4 kg aliquots. Dishes were frozen to −20 °C and transported to the lyophilization facility. The lyophilized powder (8974.18 μg SFN/g powder) was sealed in PE transportation bags and transported to the packaging facility to be filled into PE/AL storage bags. Each storage bag contained 50 μmol SFN in a 1.2 ± 0.1 g powder load. Storage bags were maintained at approximately −20 °C before dispensation to patients. The lyophilized powder was repeatedly checked for microbial contaminants. The sulforaphane powder was diluted with a small amount of water before intake to increase its bioavailability [22].

The placebo powder was prepared using spinach puree (Agro Jesenice, Zlatníky-Hodkovice, Czech Republic). The spinach puree was divided into aluminium dishes, cooled to −18 °C, lyophilized, and powdered. The placebo powder was filled into PE/AL storage bags containing 1.2 ± 0.1 g of powder load each.

### 2.4. Statistics

All analyses were completed using MedCalc version 20.010 (MedCalc Software Ltd., Ostend, Belgium) and OriginPro 8.5.0 SR1 (OriginLab Corporation, Northampton, MA, USA). Descriptive statistics were used to characterize the variables. A one-way repeated measures analysis of variances (ANOVA) followed by a Tukey post hoc test was used to determine whether three group means within each study group were different. Student’s *t*-test (unpaired two-sample test) was performed to test differences between groups at each follow-up. For comparison of nominal variables, the X^2^ test for a 2 × 2 or higher contingency table was used. *p* values less than 0.05 were considered statistically significant.

### 2.5. Ethics

All information was accessed in accordance with the applicable laws and ethical requirements for the study period concerned, and was compliant with the Declaration of Helsinki, revised in 2000. All parents/caregivers were thoroughly educated and consented at the screening visit. The study was approved by the Institutional Review Board (IRB) of the General University Hospital in Prague (Ethics Committee Approval Number: 158/19).

## 3. Results

### 3.1. Study Population

**Figure 1:**
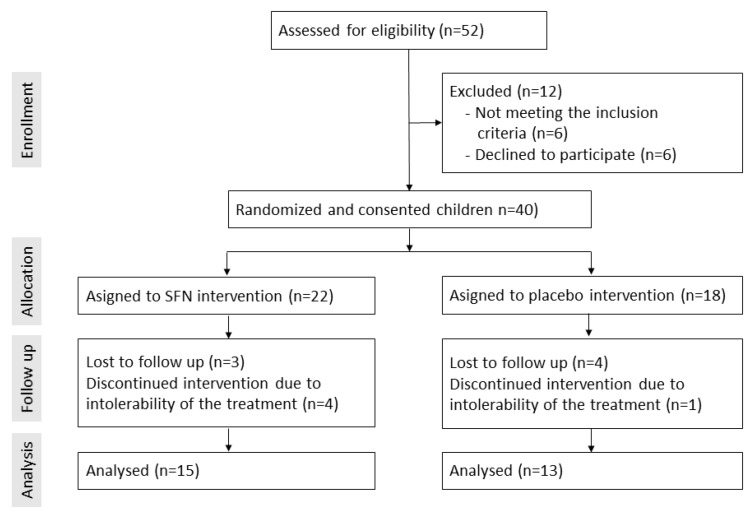
Enrollment, follow-up, and analysis of the clinical study.

A total of 40 children qualified for our study and were randomized (Figure 1). Five children dropped out due to intolerability of the preparate. Seven children were lost to follow-up and were excluded from the study analyses. Twenty-eight children completed the study, and their data were used for statistical analyses. The characteristics of the children who provided all follow-up data are shown in Table 1. All children had a diagnosis of ASD; approximately 80% were male, the mean age was 4.4 years, and there was no record of known genetic conditions or chronic disease among the children. The treatment with SFN was well-tolerated. No side or adverse effects were recorded.

### 3.2. Changes in Symptoms

**Figure 2:**
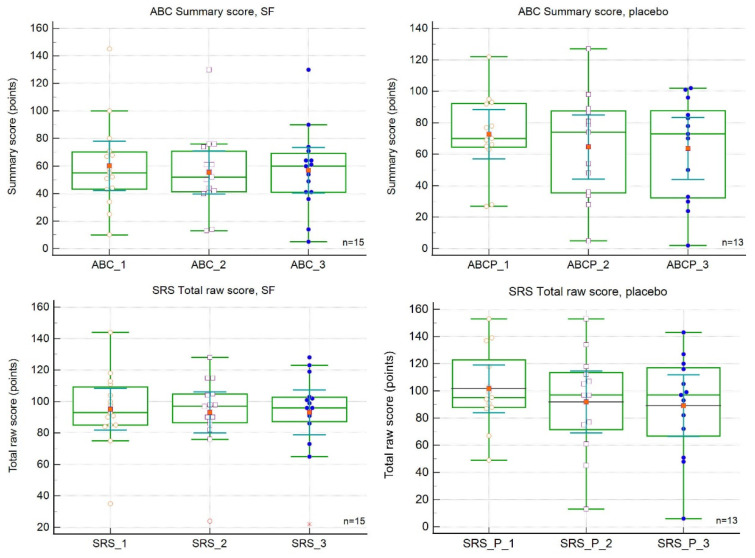
Effect of sulforaphane treatment on Aberrant Behaviour Checklist (ABC, upper panels) and Social Responsiveness Scale (SRS-2, lower panels) scores. SFN, sulforaphane group. Red dots represent score means.

The changes in SRS and ABC scores over the study period are shown in Table 2 and Table 3 and Figure 2. The baseline ABC and SRS scores did not differ between the two populations. The mean total raw scores on both measures showed improvements (decreases), but the changes were not significant. The mean ABC score improved by 12.0 points, and the mean SRS score improved by 2.0 points. Interestingly, the regression curve analysis revealed a greater magnitude of the slope for the placebo curves, indicating a greater rate of change. The mean ABC score improved by 12.0 points, and the mean SRS score improved by 2.0 points. Figure 1 demonstrates the marked improvement at the first follow-up (18 weeks) and its decline (ABC) or stop/worsening (SRS-2) at the second follow-up (36 weeks). There were no significant differences between the SFN and placebo groups at 18 or 36 weeks (Table 2 and Table 3). The differences among visits were also nonsignificant.

**Figure 3:**
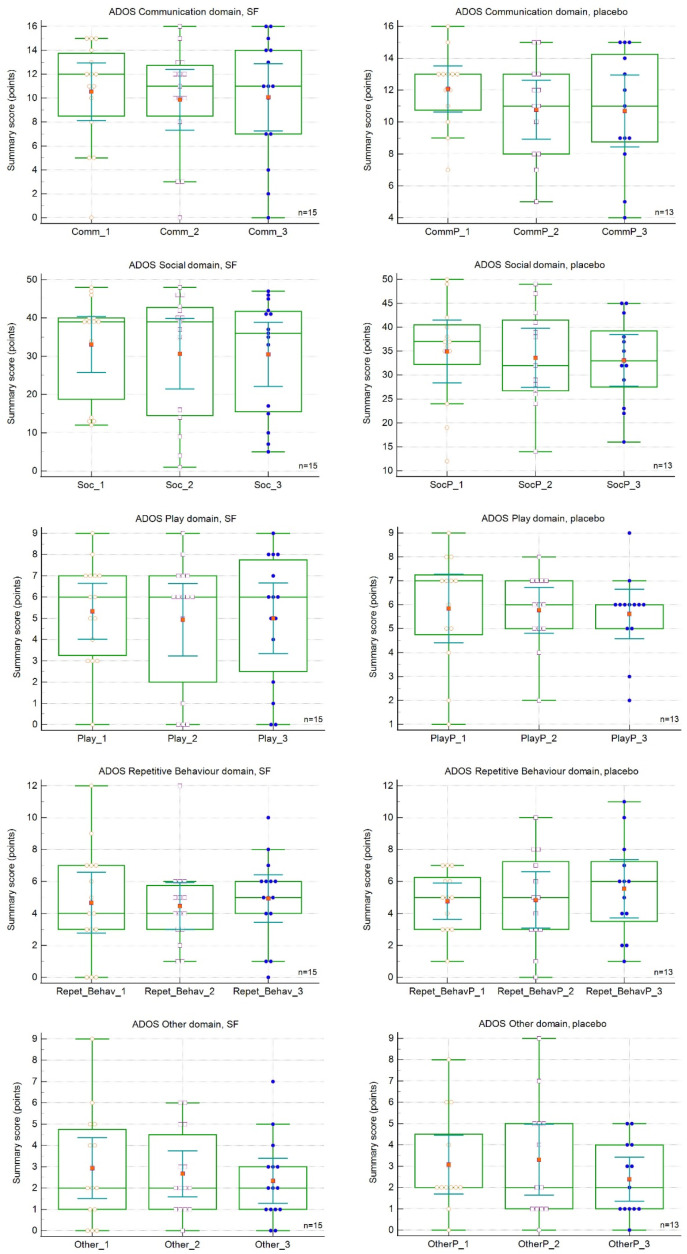
Effect of sulforaphane treatment on Autism Diagnostic Observation Schedule-2 (ADOS-2) subscale scores. SFN, sulforaphane group. Red dots represent score means.

Changes in the assessment of the ADOS-2 subscale scores are presented in Table 4 and Figure 3. The baseline and follow-up ADOS-2 scores did not differ between the two study cohorts (Table 4). There were no significant differences between the SFN and placebo PL groups at 18 or 36 weeks (Table 4), and the differences among visits were also nonsignificant. Similar to the ABS and SRS-2 surveys, the mean summary subscale scores at both visits mostly showed improvements (decreases), but the changes were nonsignificant. This trend is depicted in Figure 3.

Subjects’ scores at 18 or 36 weeks were subtracted from the same individual’s scores at the time of previous observation; differences were averaged and are presented as the means ± SEMs. *p* values indicate significant differences in total raw score means between the placebo and SFN at each intervention point.

Subjects’ scores at 18 or 36 weeks were subtracted from the same individual’s scores at the time of previous observation; differences were averaged and are presented as the means ± SEMs. *p* values indicate significant differences in summary score means between the placebo and SFN at each intervention point.

### 3.3. Comparison of Parental and Trained Professional Ratings of Children’s ASD Symptoms or Feature Development

We attempted to compare concordance and assess the eventual interchangeability of parents’ ratings of therapeutic efficacy in their children and the assessment of therapeutic efficacy performed by a trained professional. The parents’ impression scale was classified as follows: −1, the child worsened; 0, no noticeable change; and 1, the child improved somewhat [23]. At the 18-week visit, all parents of the children in the placebo group and 73% of the parents of the children in the SFN group considered their children to have improved. At the end of the study, parents’ impressions were more diversified, and the number of parents reporting no noticeable changes increased (Table 5).

Clinical psychologists evaluated the regression of behavioural symptoms in probands at the end of the study. Approximately 80% of the children in both groups did not show clinical improvement. In contrast, no children in the placebo group were classified as having no further improvement based on their parents’ impression scale ratings, whereas only one child within the SFN group was assessed as having no further improvement by their parent. We compared the two assessment techniques using the Bland–Altman plot and statistics. The line of equality is not within either the 95% CI of the mean difference or the 95% CI of the regression curve, and the bias indicates a lack of agreement between the two methods of assessment. The parents’ ratings of therapeutic efficacy and the clinical assessments of child regression indicated a significant systematic difference (H_0_: Mean = 0 with *p* = 0.0001).

## 4. Discussion

No significant clinical improvement was demonstrated in the behavioural outcome measures evaluated in ASD children aged 3–7 years treated with sulforaphane (SFN) in our study. The mean total raw scores on ABC and SRS-2 improved in the SFN and placebo groups, but none of the changes reached statistical significance. Changes in the assessment of the ADOS subscale scores also did not differ between the two study cohorts. There were no significant differences between the SFN and placebo groups at any time point or with any of the methods used. Similar to the ABS and SRS-2 surveys, the mean summary subscale scores on both visits mostly showed improvement, but the changes were also not significant.

Numerous studies have revealed that placebo effects can affect the information capability of rating scales assessed by parents or caregivers [24]. The reliability of the methods used might thus be questioned, as two parent- or caregiver-reported questionnaires (ABC and SRS-2) were collected at each visit. However, the study was double-blinded, and neither objective evidence of efficacy (ADOS-2) assessed by trained professionals reported any significant benefit of SFN. Thus, we consider this possibility marginal. A limitation of our project included its small sample size with a limited age span. Another source of weakness in this study was that the outcomes were derived from three scales only. It would be beneficial to use different scales with distinguished psychometric properties to evaluate the treatment effects more complexly. The comparability of the data was also impacted by our own unique SFN formula preparation, although the SFN content and stability have been thoroughly analysed. Other aspects, such as population heterogeneity or geographic differences in the gut microbiota composition with an impact on crosstalk between the gut–brain axis and supposed SFN-mediated reduction of oxidative stress in the brain, may be mentioned [25,26,27]. However, none of these factors should represent a decisive factor indicating the absence of an effect of SFN supplementation in our study.

Contradicting trial results provided mixed evidence of the effect of SFN [14,17,18,19]. In the first comprehensive network meta-analysis on pharmacological and dietary-supplement interventions for ASD, the authors found the efficacy of SFN inconclusive [28]. The existing evidence of the effect of SFN in autism has been gathered by studies with various designs utilizing different SFN sources and treatment durations, typically with a small sample size of variably aged subjects. Therefore, great caution must be applied when drawing comparisons between studies, as the findings might not be fully transferable to different study populations [27]. The principal work of Singh et colleagues (2014) showed marked improvements in both the ABC and the SRS scores in young men aged 13–27 years receiving oral SF during the 18-week trial [14]. Zimmerman and colleagues observed similar findings in their following clinical trials [17]. In their 36-week randomized parallel double-blind placebo-controlled clinical trial, 45 children with ASD aged 3–12 years demonstrated small but non-statistically significant effects of SF treatment evaluated with the OACIS-I. The authors also observed significant improvement measured by the ABC but not the SRS-2 [17]. Evans and Fuller [15] studied the extent of improvement in ASD patients taking SF. In a limited number of subjects followed for 28 weeks, the authors found 74 attributes (80%) of 92 improved, with significant improvements in 36 (39%) of them. Moreover, the improvements lasted for an interval of 28 weeks [15]. Similarly, in an open-label study of Bent et al. (2018), clinical testing using ABC and SRS scores in 15 children confirmed significant improvement in ABC and SRS. On the top of that, they identified 77 urinary metabolites correlated with the changes in symptoms [16]. Within the domains of social-communication difficulties (measured mainly with ABC-L/SW, Aberrant Behavior Checklist Lethargy/Social-Withdrawal subscale, and VABS-S, the Vineland Adaptive Behavior Scales Socialisation Domain), repetitive behaviours (ABC-S and CYBOCS, the Children’s Yale-Brown Obsessive Compulsive Scale), and overall core ASD symptoms (SRS and CARS, Childhood Autism Rating Scale), no clear differences between the SFN and placebo were found in children and/or adolescents [28]. Regarding the secondary outcomes of the study, SFN improved irritability measured mainly with ABC-I (Aberrant Behavior Checklist Irritability Subscale). There was also a positive trend noted for SFN in ADHD (Attention-Deficit Hyperactivity Disorder) symptom improvement measured with the ABC-H survey (Aberrant Behavior Checklist Hyperactivity/Noncompliance subscale) [28,29,30,31]. On the other hand, McGuinness and Kim reviewed five clinical trials that showed a significant positive correlation between SFN use and behavior, social, and cognitive scores [32]. Otherwise, compared to the placebo group, Momtazmanesh et al. (2020) showed in the sulforaphane group of ASD patients greater improvements the in Irritability score, Hyperactivity/Noncompliance score, and significant Time × Treatment effect for Irritability and Hyperactivity/Noncompliance. However, they did not demonstrate any difference in the Lethargy/Social Interaction score, Stereotypic Behaviour score, and Inappropriate Speech scores [33]. In a review of the current psychopharmacological treatment in ASD by Aishworiya and colleagues (2022), no significant improvement in the OACIS scores was seen in patients on SFN. The authors further reported the significant ABC improvement but not the SRS on sulforaphane vs. placebo, and significant improvements in the biomarkers including the glutathione redox status, mitochondrial respiration, inflammatory markers, and heat shock proteins on sulforaphane vs. placebo. Those improvements correlated with improvements on the ABC scale [34]. These discrepancies should be taken into account when considering SFN’s effect on ASD patients.

Previous studies have reported that parents’/caregivers’ beliefs about how a treatment will work are efficacious. Parents tend to give favourable ratings to treatments that much research has shown to be ineffective. This was also shown in the parents of our patients. The vast majority of parents reported some clinical improvement irrespective of taking the SFN or placebo in the first phase with a lesser effect in the second phase of the study (Table 5). Parental-reported improvements in their children believed to be in the treatment phase, and vice versa (their worsening when presumed to be on placebo), are well-documented [29,30]. Silva Pereira and colleagues [31] studied contrasting perspectives from professionals and parents in a sample of 136 Portuguese children aged 3–6. Parents and professionals used the Assessment Scale for Children. The authors thoroughly documented that parents evaluated their children’s development and learning more positively than professionals. The difference was marked in the field of social communication. This rating divergence was lesser in parents with higher academic qualifications [31]. We compared parents’ and professionals’ ratings of therapeutic efficacy with similar outcomes. Parents’ impressions are respected and important but should not be accepted as an objective rating. However, we suggest harnessing parents’ observations in natural daily family contexts in an effort to find a better convergence of parents’ and professionals’ assessment of children with ASD.

## 5. Conclusions

This investigation aimed to assess the effects of SFN on the reduction of ASD symptom severity. We examined a paediatric cohort of ASD patients aged three to seven years. We found no significant clinical improvement in the behavioural outcome measures evaluated in ASD children aged 3–7 years treated with sulforaphane (SFN). The evidence from this study suggests that, despite the reasonable level of knowledge of the action and metabolic pathways of SFN and proposed SFN-elicited cellular response, the clinical effects of SFN in ASD patients must be prudently evaluated. More thoughtful research is needed to better understand the true nature of the clinical benefits of SFN in the supportive treatment of ASD. Avenues for future research include large and longer-duration randomized controlled trials also considering the susceptibility to placebo effects that could provide more definitive evidence. If the debate is to be moved forwards, a better understanding of autistic spectrum disorders’ aetiology and underlying factors needs to be developed to avoid the boundless but vain hope for a miraculous treatment.

## Figures and Tables

**Table 1:** Baseline characteristics of patients.

Characteristics (Mean ± SD)	Placebo Group (*n* = 13)	SFN Group (*n* = 15)	*p* Value
Age	4.4 ± 1.5	4.6 ± 1.0	0.7475
Sex (M/F)	11/2	13/2	0.8793
Weight (kg)	19.9 ± 3.9	19.1 ± 2.7	0.5272
Medication	4/13	2/15	0.4865
Dietary supplements	1/13	2/15	0.4755
History of autistic developmental regression	10/13	12/15	0.8501

M, male; F, female; SD, standard deviation.

**Table 2:** Effect of sulforaphane treatment on Social Responsiveness Scale (SRS-2) scores.

	Total and Changes in SRS Mean Total Raw Scores		
	Time of observation (weeks)		
Scale and treatment	0	18	36
SRS			
Placebo			
Intervention point	101.5	91.8	89.5
Change	-	−9.7 ± 6.1	−2.7 ± 3.2
SFN			
Intervention point	95.1	93.1	93.1
Change	-	−2.0 ± 3.1	−0.1 ± 3.1
*p* value (between placebo and SFN)	0.5235	0.9176	0.7435

**Table 3:** Effect of sulforaphane treatment on Aberrant Behaviour Checklist (ABC) scores.

	Total and Changes in ABC Mean Summary Scores		
	Time of observation (weeks)		
Scale and treatment	0	18	36
ABC			
Placebo			
Intervention point	72.7	64.6	63.6
Change	-	−8.1 ± 6.2	−1.0 ± 3.9
SFN			
Intervention point	60.2	55.3	56.9
Change	-	−4.9 ± 4.2	1.6 ± 3.2
*p* value (between placebo and SFN)	0.2742	0.4352	0.576

**Table 4:** Effect of sulforaphane treatment on Autism Diagnostic Observation Schedule-2 (ADOS-2) scores.

	Total and Changes in ADOS Mean Summary Scores		
	Time of observation (weeks)		
Scale and treatment	0	18	36
ADOS-2 Language and communication			
Placebo			
Intervention point	12.1	10.8	10.7
Change	-	−1.3 ± 0.5	−0.1 ± 0.5
SFN			
Intervention point	10.5	9.9	10.1
Change	-	−0.7 ± 0.3	0.2 ± 0.4
*p* value (between placebo and SFN)	0.2652	0.5519	0.7165
ADOS-2 Reciprocal social interaction			
Placebo			
Intervention point	34.9	33.6	33.1
Change	-	−1.3 ± 2.1	−0.5 ± 1.4
SFN			
Intervention point	33.1	30.7	30.5
Change	-	−2.4 ± 1.6	−0.2 ± 1.1
*p* value (between placebo and SFN)	0.6907	0.5850	0.5916
ADOS-2 Play and imagination			
Placebo			
Intervention point	5.8	5.8	5.6
Change	-	−0.08 ± 0.5	−0.2 ± 0.3
SFN			
Intervention point	5.3	4.9	5.0
Change	-	−0.4 ± 0.5	0.07 ± 0.2
*p* value (between placebo and SFN)	0.5742	0.3867	0.5198
ADOS-2 Stereotyped behaviours and restricted interests			
Placebo			
Intervention point	4.8	4.8	5.5
Change	-	0.08 ± 0.6	0.7 ± 0.4
SFN			
Intervention point	4.7	4.5	4.9
Change	-	−0.2 ± 0.5	0.5 ± 0.5
*p* value (between placebo and SFN)	0.9244	0.7219	0.5791
ADOS-2 Other behaviours			
Placebo			
Intervention point	3.1	3.3	2.4
Change	-	0.2 ± 0.7	−0.9 ± 0.6
SFN			
Intervention point	2.9	2.7	2.3
Change	-	−0.3 ± 0.7	−0.3 ± 0.3
*p* value (between placebo and SFN)	0.8782	0.4788	0.9414

**Table 5:** Frequencies of parental ratings of children’s ASD symptoms or feature development. The parents’ impression scale was classified as follows: −1, the child worsened; 0, no noticeable change; and 1, the child improved somewhat. * *p* values indicate significant differences between the placebo and SFN groups.

Characteristics (Mean ± SD)	Placebo Group (*n* = 13)			SFN Group (*n* = 15)			*p* Value *
	−1	0	1	−1	0	1	
Parents’ impression (18 weeks)	0/13	0/13	13/13	1/15	3/15	11/15	0.1324
Parents’ impression (36 weeks)	0/13	7/13	6/13	1/15	5/15	9/15	0.4066
Parents’ impression at 18 weeks vs. 36 weeks	*p* = 0.0028 *			*p* = 0.8940			-

## Data Availability

The data are available upon reasonable request to the corresponding author.
